# Supplementary material for: Carbon Quantum Dots Bridged TiO2/CdIn2S4 toward Photocatalytic Upgrading of Polycyclic Aromatic Hydrocarbons to Benzaldehyde
Source: Molecules. 2022 Oct 27;27(21):7292. doi: 10.3390/molecules27217292 (PMC9653999; doi:10.3390/molecules27217292)
Supplement: Supplementary file 1 [file molecules-27-07292-s001.zip › molecules-1970602-supplementary.pdf]

Supporting Information

Carbon quantum dots bridged $TiO_2/CdIn_2S_4$ toward photocatalytic upgrading of polycyclic aromatic hydrocarbons to benzaldehyde

Jiangwei Zhang[12#], Fei Yu[1#], Xi Ke[3], He Yu[1], Peiyuan Guo[1], Lei Du[2], Menglong Zhang[1*] and Dongxiang Luo[2*]

[1]Institute of hydrogen energy for carbon peaking and carbon neutralization, School of Semiconductor Science and Technology, South China Normal University, Foshan, 528225, P.R. China

[2]School of Chemistry and Chemical Engineering/Huangpu Hydrogen Innovation Center, Guangzhou University, Guangzhou 510006, PR China

[3]Great Bay Area Branch of Aerospace Information Research Institute, Chinese Academy of Sciences, Guangzhou 510530, China

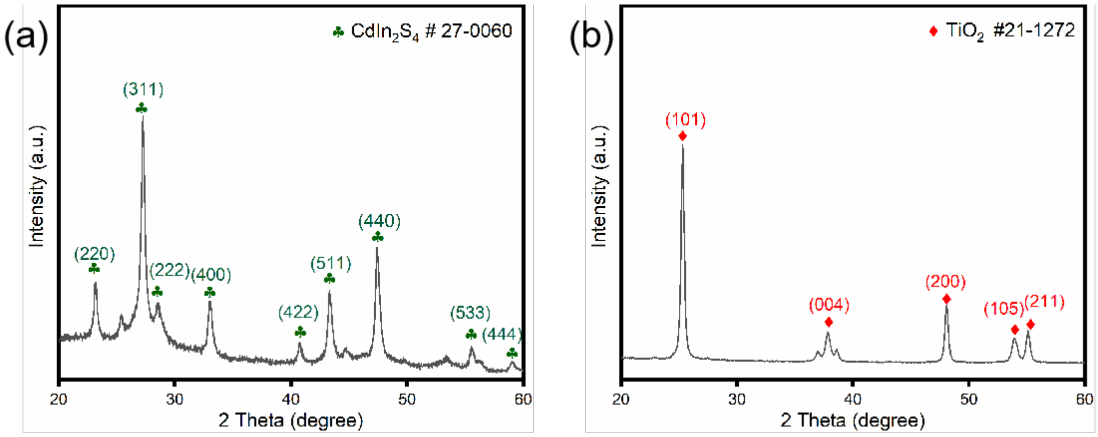

Figure S1: XRD of (a) bare $CdIn_2S_4$ and (b) bare TiO_2

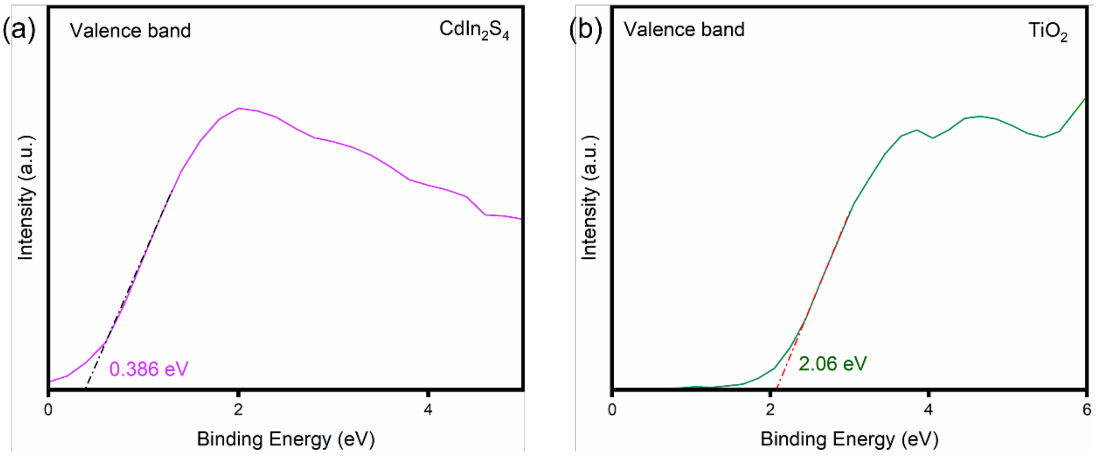

Figure S2: The valance band edges of (a) CdIn2S4 and (b) TiO2.

After obtaining XPS valence band data, the following two formulas shall be used to convert the data into NHE[1] and RHE[2], respectively:

$$E_{VB,NHE} = \varphi + E_{VB,XPS} - 4.44 \quad (1)$$

$$E_{NHE} = E_{NHE} + 0.0591PH \quad (2)$$

where the φ represent work function of the instrument (4.2 eV).

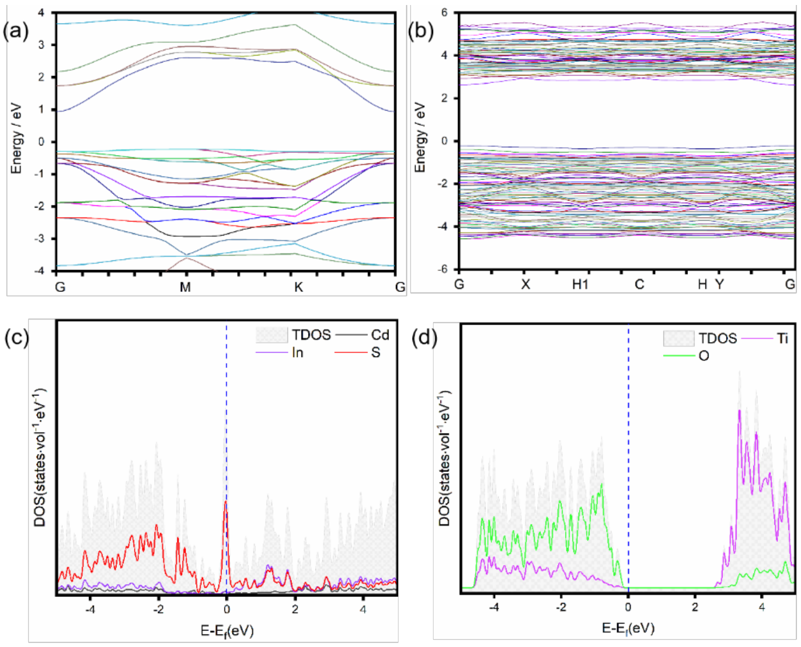

Figure S3: (a-b) the band structure of bare $CdIn_2S_4$ bulk and bare TiO_2 bulk, (c-d) the density of states for bare $CdIn_2S_4$ bulk and bare TiO_2 bulk

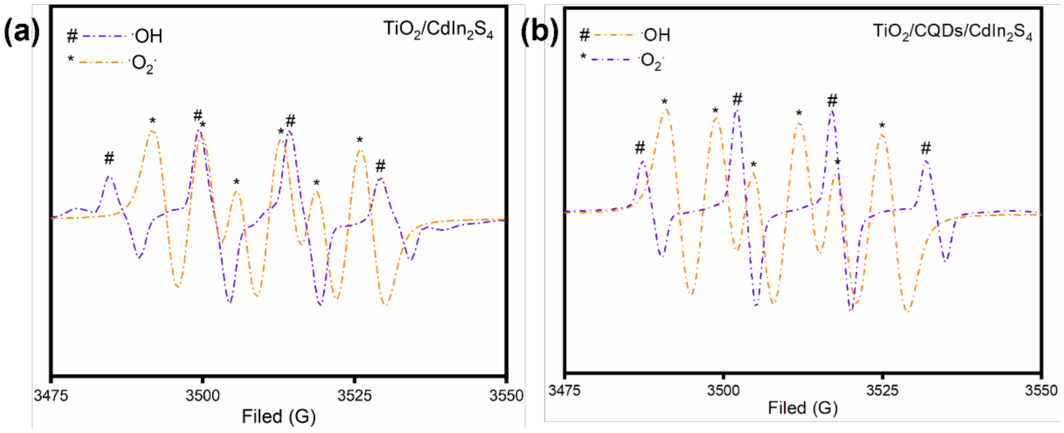

Figure S4: EPR spectrum of (a) $TiO_2/CdIn_2S_4$ and (b) $TiO_2/CQDs/CdIn_2S_4$.

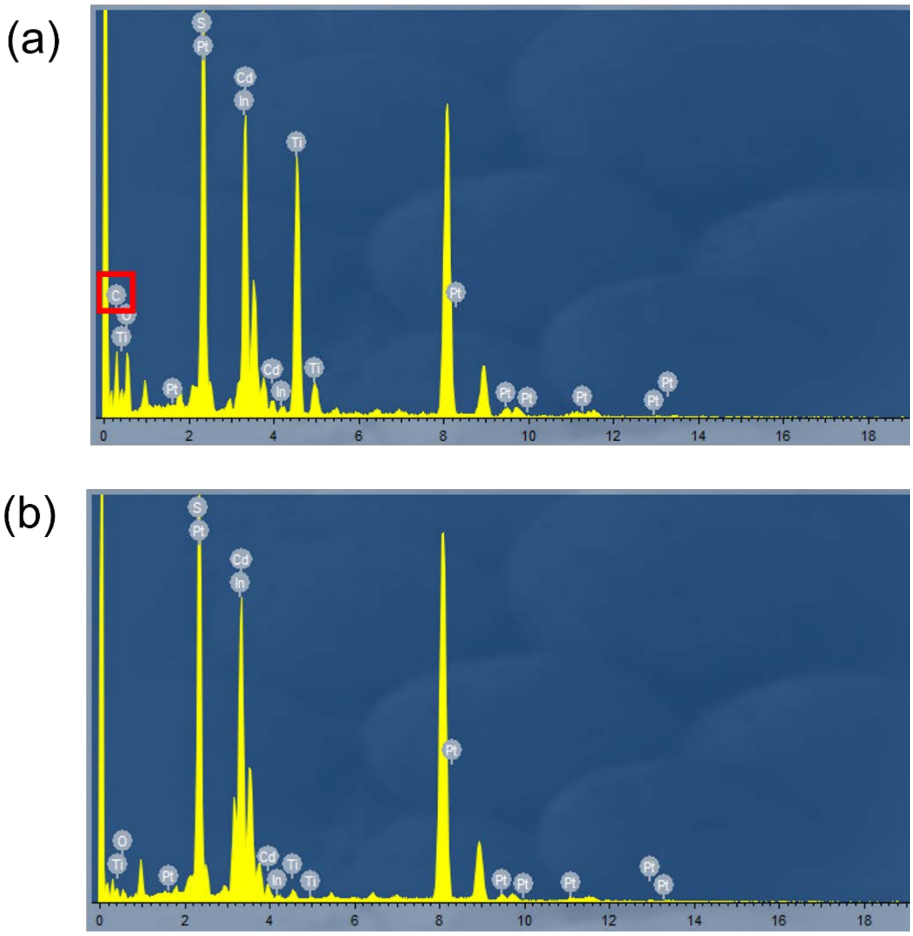

Figure S5: The elemental composition of (a) $TiO_2/CQDs/CdIn_2S_4$ and (b) $TiO_2/CdIn_2S_4$ analyzed by TEM-EDS.

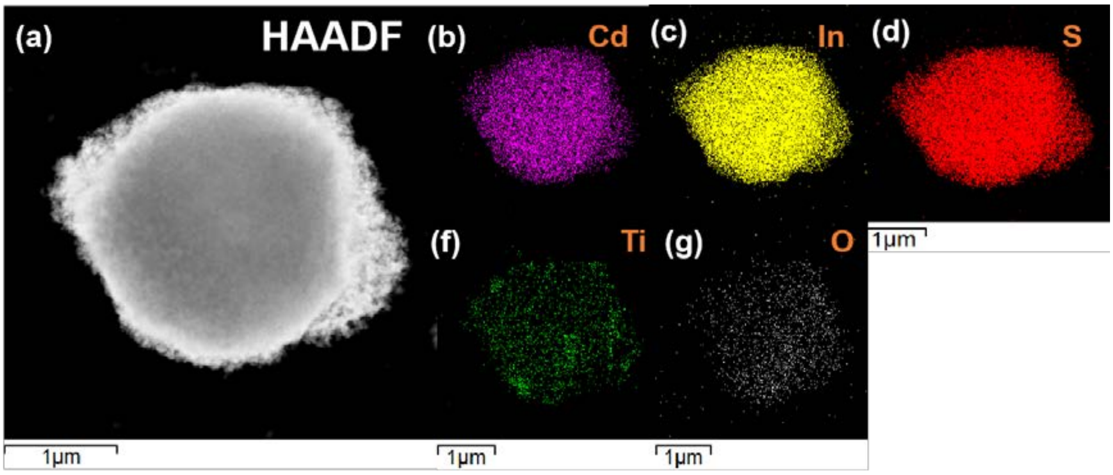

Figure S6: (a) HAADF TEM image of $TiO_2/CdIn_2S_4$ heterostructure, (b-g) respective elemental mapping of Cd, In, S, Ti and O.

Table S1. Relative atomic content of TiO_2/CQDs/$CdIn_2S_4$ and TiO_2/$CdIn_2S_4$.

Sample	C/%	Cd/%	In/%	S/%	Ti/%	O/%	Pt/%
TiO_2/CQDs/$CdIn_2S_4$	19.27	2.32	20.75	28.98	17.95	10.05	0.69
TiO_2/$CdIn_2S_4$	0	11.74	32.44	51.93	0.91	1.99	0.99

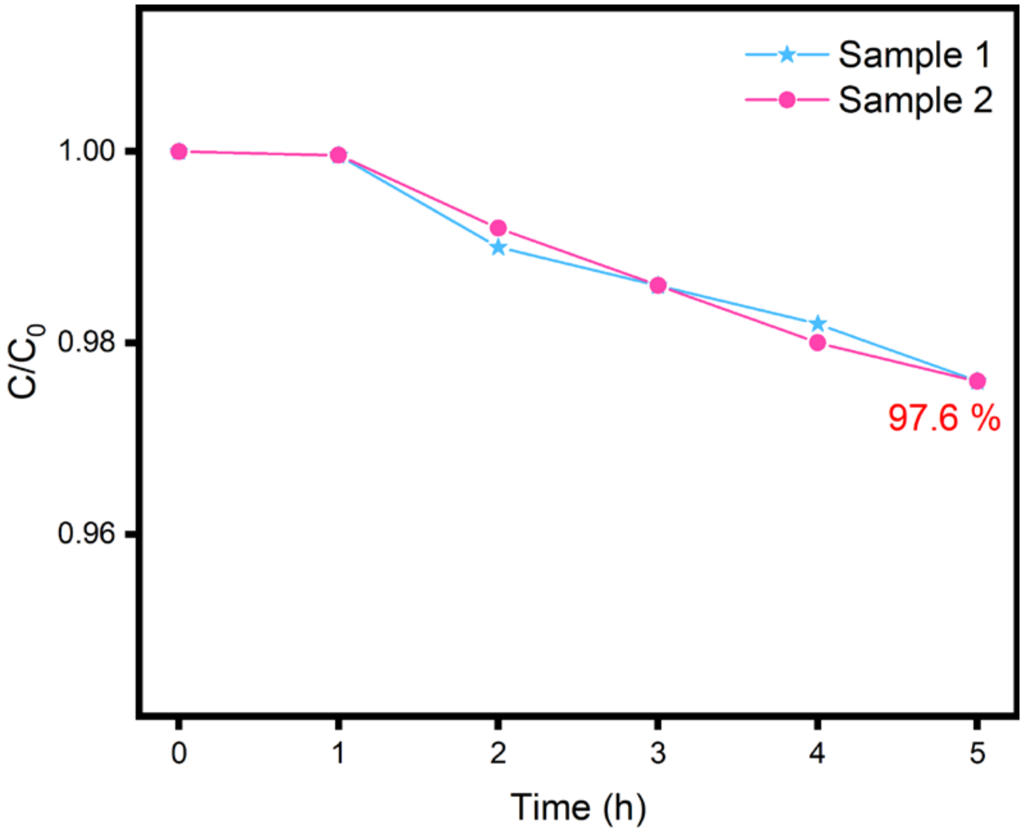

Figure S7: Stability of PAHs degradation of TiO_2/CQDs/$CdIn_2S_4$.

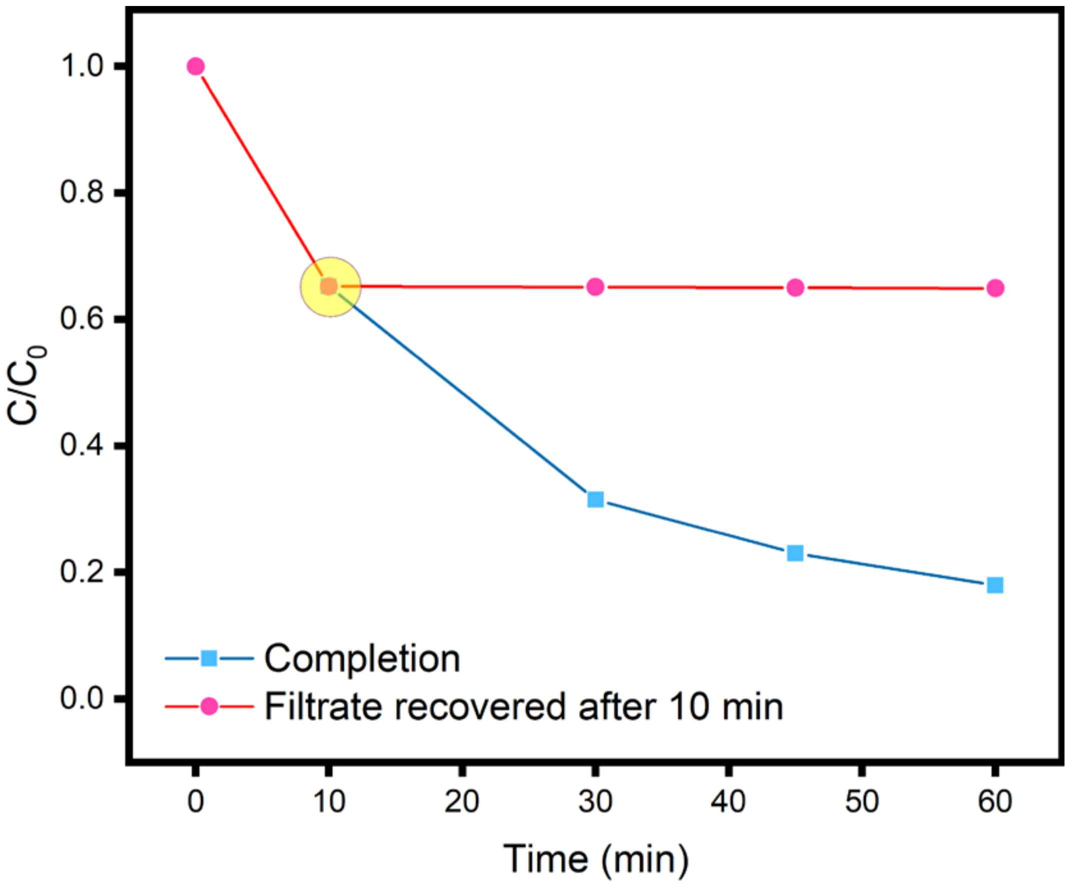

Figure S8: Hot filtration test of TiO_2/CQDs/$CdIn_2S_4$.

1. Li, X.; Kang, B.; Dong, F.; Zhang, Z.; Luo, X.; Han, L.; Huang, J.; Feng, Z.; Chen, Z.; Xu, J.; et al. Enhanced photocatalytic degradation and H2/H2O2 production performance of S-pCN/WO2.72 S-scheme heterojunction with appropriate surface oxygen vacancies. *Nano Energy* **2021**, *81*, 105671, doi:10.1016/j.nanoen.2020.105671.
2. Li, Y.; Wang, K.; Huang, D.; Li, L.; Tao, J.; Ghany, N.A.A.; Jiang, F. CdxZn1-xS/Sb2Se3 thin film photocathode for efficient solar water splitting. *Applied Catalysis B: Environmental* **2021**, *286*, doi:10.1016/j.apcatb.2020.119872.
